# Validation of the OrthoGnathicAnalyser 2.0—3D accuracy assessment tool for bimaxillary surgery and genioplasty

**DOI:** 10.1371/journal.pone.0246196

**Published:** 2021-01-26

**Authors:** Frank Baan, Juliana F. Sabelis, Ruud Schreurs, Gert van de Steeg, Tong Xi, Tom C. T. van Riet, Alfred G. Becking, Thomas J. J. Maal

**Affiliations:** 1 Radboudumc 3DLab The Netherlands, Radboud University Medical Center, Radboud Institute for Health Sciences, Nijmegen, The Netherlands; 2 Department of Oral and Maxillofacial Surgery, Radboud University Medical Center, Nijmegen, The Netherlands; 3 Department of Oral and Maxillofacial Surgery, Amsterdam UMC Location AMC and Academic Center for Dentistry Amsterdam (ACTA), University of Amsterdam, Amsterdam, The Netherlands; 4 Department of Oral and Maxillofacial Surgery, MKA Kennemer & Meer, Haarlem, The Netherlands; Navodaya Dental College and Hospital, INDIA

## Abstract

Orthognathic surgery is a widely performed procedure to correct dentofacial deformities. Virtual treatment planning is an important preparation step. One advantage of the use of virtual treatment planning is the possibility to assess the accuracy of orthognathic surgery. In this study, a tool (OrthoGnathicAnalyser 2.0), which allows for quantification of the accuracy of orthognathic surgery, is presented and validated. In the OrthoGnathicAnalyser 2.0 the accuracy of the osseous chin can now be assessed which was not possible in the earlier version of the OrthoGnathicAnalyser. 30 patients who underwent bimaxillary surgery in combination with a genioplasty were selected from three different centers in the Netherlands. A pre-operative (CB)CT scan, virtual treatment planning and postoperative (CB)CT scan were required for assessing the accuracy of bimaxillary surgery. The preoperative and postoperative (CB)CT scans were aligned using voxel-based matching. Furthermore, voxel-based matching was used to align the pre-operative maxilla, mandible and rami towards their postoperative position whereas surface-based matching was used for aligning the pre-operative chin towards the postoperative position. The alignment resulted in a transformation matrix which contained the achieved translations and rotations. The achieved translations and rotations can be compared to planning values of the virtual treatment plan. To study the reproducibility, two independent observers processed all 30 patients to assess the inter-observer variability. One observer processed the patients twice to assess the intra-observer variability. Both the intra- and inter-observer variability showed high ICC values (> 0.92) and low measurement variations (< 0.673±0.684mm and < 0.654±0.824°). The results of this study show that the OrthoGnathicAnalyser 2.0 has an excellent reproducibility for quantification of skeletal movements between two (CB)CT scans.

## Introduction

In orthognathic surgery, suboptimal facial appearance and function may be improved by correcting dentofacial deformities [1]. Three-dimensional (3D) imaging has enhanced the potential and accuracy of the orthognathic surgery workflow [2]. The introduction of cone-beam computed tomography (CBCT) in combination with virtual imaging software enables diagnostics, planning and evaluation in 3D. This has improved quantification of, formerly difficult to measure, characteristics of dentofacial deformities. These include rotations in the axial plane (yaw) or frontal plane (roll or occlusal cant) [3]. Additive manufactured occlusal splints are based on a virtual surgical planning (VSP) and are used to accurately execute the VSP during surgery [4]. These new 3D techniques have led to more predictable postoperative outcomes and a reduction of surgical error [5].

Similar to VSP, postoperative accuracy of the performed surgery should be evaluated in 3D. The result of orthognathic surgery was traditionally assessed in two dimensions, using pre- and postoperative lateral radiographs [6]. Contemporary software enables automatic matching, also called registration, of two 3D imaging datasets. Voxel-based matching (VBM) is the registration method of preference, due to its higher accuracy and user independency [7]. In this technique, the two volumes of interest (VOI) are aligned by maximizing the overlap of the greyscale values of the individual voxels [8]. After aligning the VOI, the translations and rotations in the sagittal, coronal and axial planes (six degrees of freedom) realized by the orthognathic surgery, can be computed and compared to the VSP [9, 10]. The systematic review of Gaber et al. [7], has reviewed several 3D postoperative assessment methods of virtually planned orthognathic surgery. The OrthoGnathicAnalyser (OGA), as described in our previous study [11], was identified as the 3D assessment tool of choice, due to the application of VBM and the semi-automatic approach. Over time, the OGA has already been applied in large clinical studies [12, 13], demonstrating its clinical applicability.

After validation of the first version, the development of the OGA continued and has resulted in OGA 2.0. While the former version only enabled analysis of the mandible, maxilla, and the ramus, the new version also allows analysis of the chin segment. In addition, the efficiency of the workflow has been improved, requiring less manual interaction and computing time. The software is compatible with various VSP software, such as IPS CaseDesigner (KLS Martin Group, Tuttlingen, Germany) and Dolphin 3D (Dolphin Imaging & Management Solutions, Chatsworth, USA). The purpose of this study was to present and validate the new version of the OGA (2.0) in patients who underwent bimaxillary surgery in combination with a genioplasty. Because different centers use different imaging protocols and hardware from different manufacturers for their preoperative and postoperative imaging, a multicenter approach was chosen to assess the robustness of the software tool.

## Materials & methods

### Workflow of OrthoGnathicAnalyser 2.0

**Fig 1:**
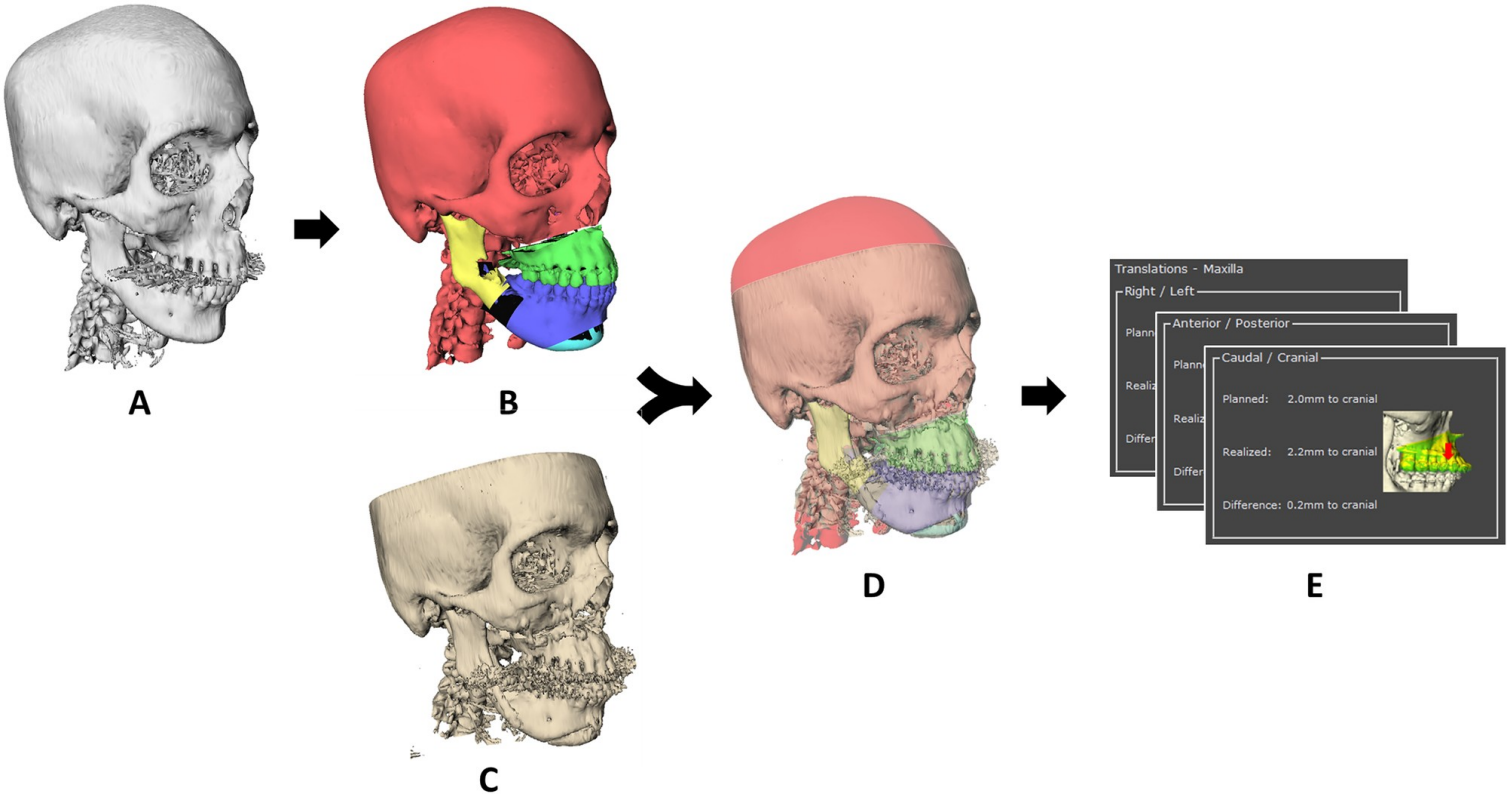
Global overview of the workflow of OrthoGnathicAnalyser 2.0. A) Preoperative (CB)CT scan of the patient. B) Virtually planned 3D models. C) Postoperative (CB)CT scan. The postoperative (CB)CT scan is voxel-based matched to the preoperative (CB)CT scan. Individual segments of the preoperative (CB)CT scan are matched to the postoperative (CB)CT scan. D) Overlap of the postoperative (CB)CT scan and planned STL models. E) planned, realized and difference of the three translation and three rotation parameters are computed for the maxilla, mandible, chin, left and right ramus.

The workflow of OGA 2.0 was based on the workflow described in the previous article [11] and is illustrated in Fig 1. In preparation for the surgery, the acquisition of a preoperative (CB)CT scan is required. This scan was used for the virtual planning of the subject with planning software. After surgery, a postoperative (CB)CT scan was acquired.

The assessment of discrepancy between VSP and postoperative outcome was performed semi-automatically, using the OGA module which was implemented in the in-house developed 3D analysis software called 3DMedX (version 1.2.4.1, 3D lab Radboudumc, Nijmegen, the Netherlands). 3DMedX is a standalone software tool based on the C++ OpenInventor Toolkit (version 9.9.14, Thermo Fisher Scientific, Waltham, Massachusetts, USA). To start the analysis with OGA, the raw preoperative and postoperative (CB)CT scans (in Digital Imaging and Communications in Medicine (DICOM) format) were imported in the software. From the VSP the following files were necessary: the original and planned 3D models (as Standard Tessellation Language (STL) files) and a transformation matrix (in extensible markup language (XML) format). The transformation matrix described the transformation of the virtual models to the natural head position (NHP) on which the VSP was based. When no transformation matrix was available, the software provided a wizard-tool to identify the NHP.

Next, the user was asked to indicate four rotation points, which were used as reference points for the calculation of translations and rotations in subsequent analyses. The first point was the upper incisor point, defined as the most mesial point on the incisal edge of element 11. The second point was pogonion, as described by Swennen et al [14]. The third and fourth points were the most cranial points of the left and right condylar head. The upper incisor point was utilized as the origin (and thus rotation point) to align the 3D models to NHP.

To compute the six degrees of freedom in VSP, the preoperative STL models were automatically matched to the planned STL models using surface-based matching (SBM). The resulting transformation matrix was calculated to represent the planned rotations and translations around the four previously indicated rotation points.

In the next steps, voxel-based matching (VBM) was used to register the individual bony segments. In VBM, a region of interest (ROI) in both scans is selected, which will be subsequently aligned based on the greyscale values [15]. First, the pre- and postoperative (CB)CT scans are aligned based on the ROI, unaffected by surgery, which consisted of the anterior cranial base, zygomatic arches and forehead [16]. For the maxilla, mandible and the left and right ramus, ROI boxes were selected to match the osteotomized bone segments. The transformation matrices, describing the translations and rotations from the preoperative models to the postoperative models, were recorded.

**Fig 2:**
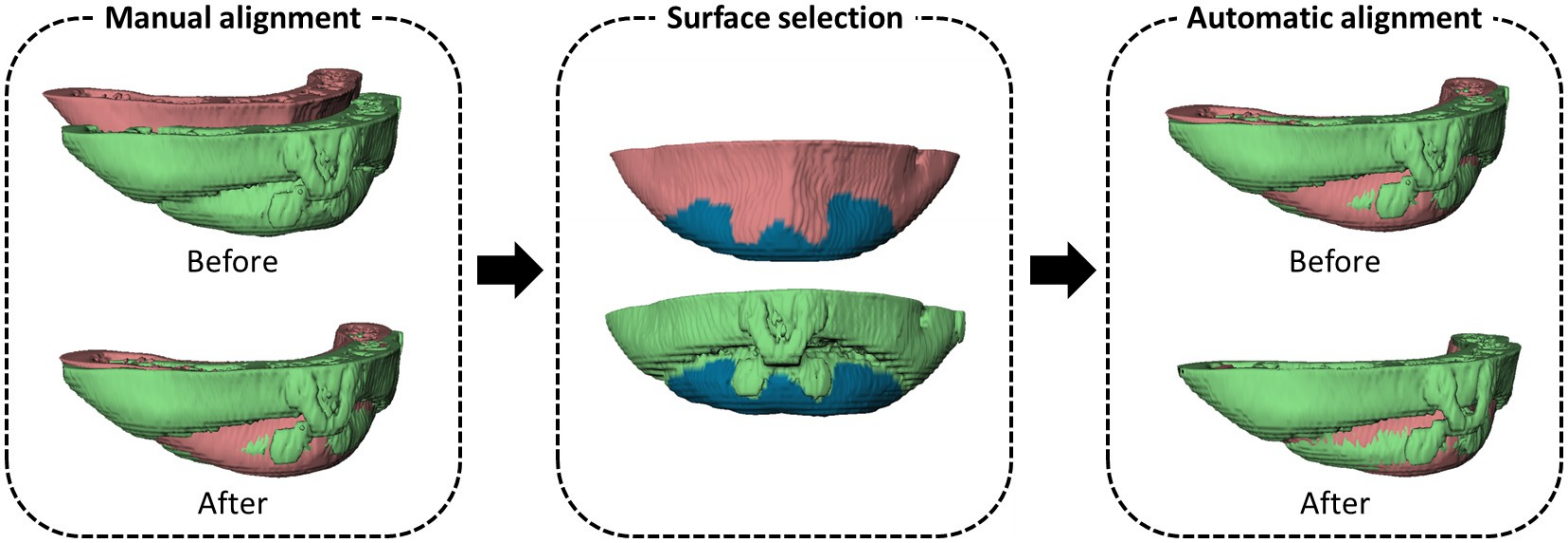
Surface-based matching of the chin. The preoperative chin model (red) was first manually aligned to the postoperative chin model (green). The user needed to select the surface on both models (blue). The selected surface will be used for the automatic surface-based matching.

For registration of the chin segment, SBM was implemented instead of VBM (see Fig 2). Surface models representing the chin were generated from the preoperative and postoperative DICOM datasets. The preoperative and postoperative chin segments are roughly aligned manually, after which SBM was performed by using the unaltered caudal part of the chin, excluding the area of osteosynthesis plate. The transformation matrix obtained after SBM of the preoperative model on the postoperative model was recorded.

The resulting transformation matrices were calculated to represent the surgically achieved rotations and translations around the four previously indicated rotation points for each segment. Finally, the differences between the planned and achieved movement of each segment in six degrees of freedom (translation and rotation in sagittal, coronal and axial plane) were calculated. For the chin evaluation, the difference between the planned movement of the chin and its postoperative position was calculated and corrected for the postoperative mandibular position. This excluded the potential mandibular error from the accuracy result of the chin. For the rami, only rotations were computed, assuming the condylar heads were not translated. (A video is available showcasing the workflow of the OrthoGnathicAnalyser2.0).

### Validation study

Thirty subjects were enrolled in this multicenter retrospective validation study, in three centers: Amsterdam University Medical Center (UMC), Location AMC, MKA Kennemer & Meer, location Haarlem and Radboud University Nijmegen Medical Center. Per center, ten subjects with dentofacial deformities who underwent bimaxillary surgery in combination with a genioplasty between 2016 and 2020 were considered for inclusion. Availability of pre- and postoperative (CB)CT data was required. Exclusion criteria were the use of different imaging modalities (i.e. a preoperative CT scan with postoperative CBCT scan or vice versa), previous history of surgery in the maxillomandibular region and high complex cases such as multi-piece Le Fort I or cleft lip and palate cases. Prior to data analysis, all subject data were anonymized. The study was approved by the local ethics committee of Amsterdam UMC, location AMC (W20_127). All patients signed an informed consent at the start of treatment.

#### Image acquisition

The preoperative scan and postoperative scan were acquired according to the clinical protocol of the individual centers. The dental arches were recorded by making a CBCT scan of dental casts. Image acquisition parameters are described in Table 1.

**Table 1:** Scanning details per center.

	Radboudumc	Amsterdam UMC		MKA Kennemer&Meer
	CBCT	CT	CBCT	CBCT
System	Imaging Sciences International I-CAT 17–19	Siemens SOMATOM Force	Planmeca ProMax	Vatech PCT-90LH
Protocol	Extended Height	Face	Skull	EzDent
Potential (kV)	120	120	96	106–108
mA	5	360	10	6
FOV	17 x 23 cm	24 x 24 cm	23 x 25 cm	21 x 21 cm
Scanning time	1.0 x 17.8 sec	1.0 x 1.0 sec	2.0 x 12.0 sec	1.0 x 18.0 sec
Voxel size	0.30 mm x 0.30 mm x 0.30 mm	0.47 mm x 0.47 mm x 10.00 mm	0.40 mm x 0.40 mm x 0.40 mm	0.40 mm x 0.40 mm x 0.40 mm

CBCT = cone beam computed tomography, CT = computed tomography, FOV = field of view, kV = kilovoltage, mA = milliampere

#### Surgery planning

All cases were virtually planned in IPS CaseDesigner, version 2.0.4.2 (KLS Martin Group, Tuttlingen, Germany). A 3D virtual hard-tissue and soft-tissue model were rendered and oriented in the NHP of the subject. The maxilla, mandible, chin and rami were repositioned towards their desired position. The required 3D models and transformation matrix were exported.

#### Clinical validation and evaluation

Two independent observers (FB and JS) analyzed the (CB)CT data sets of all cases in order to validate OGA 2.0. To determine inter-observer variability, both observers performed the OGA workflow for each subject independently. For intra-observer variability, one of the observers repeated the workflow on all cases a second time and in a random order, with an interval of at least two weeks between both assessments.

#### Statistical analysis

The absolute inter-observer and intra-observer difference was calculated. One-way multivariate analysis of variance (MANOVA) was used to determine statistical differences between centers. For the evaluation of the inter-observer and intra-observer correlation and agreement, the intra-class correlation coefficient (ICC) was calculated with two-way random and two-way mixed models respectively. Statistical data analyses were performed with IBM SPSS software, version 26.0 (IBM Corp., Armonk, NY, USA).

## Results

A total of thirty subjects (ten subjects per participating center) were included in this study. During data analysis, four subjects of the Amsterdam UMC and one subject of the MKA Kennemer&Meer had to be excluded because of motion artefacts (n = 2), corrupt DICOM data (n = 2) or incorrect field of view (n = 1). This resulted in a study population of 25 subjects. The demographics of the population are presented in Table 2.

**Table 2:** Demographics of the subjects.

	*Amsterdam UMC*	*MKA Kennemer&Meer*	*Radboudumc*	*Total*
Number of subjects	6	9	10	25
Gender (M/F)	3/3	6/3	3/7	12/13
Mean age at surgery (years)	39	27	29	31

### Validation of OGA 2.0

The inter-observer and intra-observer intraclass correlation coefficients (ICC) and the mean differences for the maxilla, mandible, chin, left and right ramus are reported in Tables 3–6 respectively.

**Table 3:** Intra-observer and inter-observer intraclass correlation coefficients (ICC) and mean differences for measurements of the maxilla.

			*Inter-observer*		*Intra-observer*	
			*ICC*	*Mean difference (±SD)*	*ICC*	*Mean difference (±SD)*
Translation (mm)	RL	Center 1	0.996	0.064 (±0.062)	0.992	0.103 (±0.054)
		Center 2	0.999	0.045 (±0.042)	0.997	0.082 (± 0.050)
		Center 3	0.992	0.055 (±0.029)	0.992	0.048 (± 0.041)
		**Mean**	**0.996**	**0.055 (±0.042)**	**0.994**	**0.074 (±0.052)**
	AP	Center 1	0.938	0.251 (±0.245)	0.991	0.109 (± 0.077)
		Center 2	0.996	0.146 (±0.119)	0.997	0.097 (± 0.132)
		Center 3	0.991	0.089 (±0.113)	0.996	0.073 (± 0.059)
		**Mean**	**0.975**	**0.147 (±0.165)**	**0.995**	**0.085 (±0.091)**
	SI	Center 1	0.880	0.290 (±0.320)	0.973	0.168 (± 0.114)
		Center 2	0.934	0.289 (±0.310)	0.960	0.242 (± 0.261)
		Center 3	0.954	0.197 (±0.166)	0.961	0.176 (± 0.166)
		**Mean**	**0.923**	**0.261 (±0.259)**	**0.965**	**0.202 (±0.195)**
Rotation (degrees)	Roll	Center 1	0.988	0.076 (±0.109)	0.990	0.105 (± 0.052)
		Center 2	0.963	0.168 (±0.176)	0.961	0.174 (±0.186)
		Center 3	0.945	0.175 (±0.088)	0.960	0.140 (±0.096)
		**Mean**	**0.965**	**0.152 (±0.134)**	**0.970**	**0.144 (±0.130)**
	Pitch	Center 1	0.984	0.583 (±0.643)	0.997	0.332 (±0.111)
		Center 2	0.914	0.584 (±0.815)	0.904	0.526 (±0.860)
		Center 3	0.975	0.259 (±0.273)	0.972	0.275 (±0.273)
		**Mean**	**0.958**	**0.460 (±0.614)**	**0.958**	**0.378 (±0.550)**
	Yaw	Center 1	0.986	0.122* (±0.043)	0.988	0.092 (±0.059)
		Center 2	0.998	0.066* (±0.035)	0.997	0.066 (±0.070)
		Center 3	0.994	0.076 (±0.273)	0.997	0.055 (±0.030)
		**Mean**	**0.993**	**0.084 (±0.047)**	**0.994**	**0.065 (±0.054)**

SD = standard deviation, RL = right-left, AP = anteroposterior, SI = superoinferior. Center 1 = Amsterdam UMC, Center 2 = MKA Kennemer&Meer, Center 3 = Radboudumc,

* = statistically significant difference.

**Table 4:** Intra-observer and inter-observer differences and intraclass correlation coefficients (ICC) for measurements of the mandible.

			*Inter-observer*		*Intra-observer*	
			*ICC*	*Mean difference (±SD)*	*ICC*	*Mean difference (±SD)*
Translation (mm)	RL	Center 1	0.997	0.110 (±0.095)	0.997	0.114 (±0.059)
		Center 2	0.995	0.099 (±0.085)	0.997	0.075 (±0.064)
		Center 3	0.993	0.115 (±0.075)	0.998	0.062 (±0.039)
		**Mean**	**0.995**	**0.107 (±0.082)**	**0.997**	**0.078 (±0.056)**
	AP	Center 1	0.995	0.237 (±0.201)	0.999	0.124 (±0.077)
		Center 2	1.00	0.081 (±0.064)	1.00	0.057 (±0.044)
		Center 3	0.993	0.147 (±0.129)	0.997	0.108 (±0.074)
		**Mean**	**0.996**	**0.147 (±0.143)**	**0.999**	**0.091 (±0.070)**
	SI	Center 1	0.999	0.242 (±0.194)	0.999	0.270 (±0.246)
		Center 2	0.999	0.116 (±0.097)	0.997	0.173 (±0.140)
		Center 3	0.983	0.311 (±0.272)	0.996	0.147 (±0.124)
		**Mean**	**0.994**	**0.226 (±0.220)**	**0.997**	**0.192 (±0.166)**
Rotation (degrees)	Roll	Center 1	0.981	0.163 (±0.151)	0.992	0.105 (±0.074)
		Center 2	0.929	0.330 (±0.557)	0.997	0.088 (±0.076)
		Center 3	0.943	0.129 (±0.118)	0.955	0.166 (±0.118)
		**Mean**	**0.951**	**0.228 (±0.352)**	**0.981**	**0.122 (±0.100)**
	Pitch	Center 1	0.993	0.638 (±0.543)	0.994	0.570 (±0.588)
		Center 2	0.994	0.266 (±0.299)	0.996	0.242 (±0.184)
		Center 3	0.975	0.475 (±0.260)	0.990	0.303 (±0.121)
		**Mean**	**0.987**	**0.392 (±0.293)**	**0.993**	**0.343 (±0.332)**
	Yaw	Center 1	0.993	0.142 (±0.228)	1.00	0.031 (±0.026)
		Center 2	0.997	0.103 (±0.137)	0.999	0.068 (±0.046)
		Center 3	0.988	0.129 (±0.064)	0.995	0.081 (±0.039)
		**Mean**	**0.993**	**0.126 (±0.140)**	**0.998**	**0.066 (±0.042)**

SD = standard deviation, RL = right-left, AP = anteroposterior, SI = superoinferior. Center 1 = Amsterdam UMC, Center 2 = MKA Kennemer&Meer, Center 3 = Radboudumc.

**Table 5:** Intra-observer and inter-observer differences and intraclass correlation coefficients (ICC) for measurements of the chin.

			*Inter-observer*		*Intra-observer*	
			*ICC*	*Mean difference (±SD)*	*ICC*	*Mean difference (±SD)*
Translation (mm)	RL	Center 1	0.986	0.230 (±0.231)	0.999	0.083 (±0.043)
		Center 2	0.968	0.257 (±0.281)	0.921	0.296 (±0.478)
		Center 3	0.933	0.168 (±0.113)	0.979	0.095 (±0.050)
		**Mean**	**0.962**	**0.223 (±0.210)**	**0.966**	**0.169 (±0.301)**
	AP	Center 1	0.974	0.197 (±0.215)	0.955	0.274 (±0.268)
		Center 2	0.958	0.238 (±0.219)	0.994	0.160 (±0.110)
		Center 3	0.981	0.240 (±0.108)	0.993	0.113 (±0.093)
		**Mean**	**0.971**	**0.213 (±0.158)**	**0.981**	**0.150 (±0.137)**
	SI	Center 1	0.980	0.299 (±0.177)	0.991	0.237 (±0.101)
		Center 2	0.968	0.144 (±0.218)	0.970	0.170 (±0.184)
		Center 3	0.979	0.301 (±0.285)	0.998	0.107 (±0.087)
		**Mean**	**0.976**	**0.251 (±0.245)**	**0.986**	**0.160 (±0.140)**
Rotation (degrees)	Roll	Center 1	0.980	0.199 (±0.252)	0.995	0.112 (±0.102)
		Center 2	0.967	0.415 (±0.611)	0.962	0.468 (±0.684)
		Center 3	0.99	0.213 (±0.187)	0.995	0.154 (±0.134)
		**Mean**	**0.979**	**0.285 (±0.410)**	**0.984**	**0.267 (±0.444)**
	Pitch	Center 1	0.938	1.057 (±0.629)	0.941	0.907 (±0.638)
		Center 2	0.898	0.681 (±1.225)	0.825	0.858 (±1.669)
		Center 3	0.995	0.505 (±0.442)	0.999	0.250 (±0.154)
		**Mean**	**0.944**	**0.654 (±0.824)**	**0.922**	**0.604 (±1.075)**
	Yaw	Center 1	0.994	0.395 (±0.343)	0.999	0.135 (±0.106)
		Center 2	0.982	0.348 (±0.512)	0.971	0.411 (±0.658)
		Center 3	0.968	0.311 (±0.182)	0.986	0.209 (±0.105)
		**Mean**	**0.981**	**0.345 (±0.362)**	**0.985**	**0.264 (±0.414)**

SD = standard deviation, RL = right-left, AP = anteroposterior, SI = superoinferior. Center 1 = Amsterdam UMC, Center 2 = MKA Kennemer&Meer, Center 3 = Radboudumc.

**Table 6:** Intra-observer and inter-observer differences and intraclass correlation coefficients (ICC) for measurements of the left and right ramus per center.

			*Inter-observer*		*Intra-observer*	
			*ICC*	*Mean difference (±SD)*	*ICC*	*Mean difference (±SD)*
Left ramus Rotation (degrees)	Auto	Center 1	0.947	0.954 (±0.830)	0.986	0.599 (±0.420)
		Center 2	0.952	0.331 (±0.269)	0.971	0.410 (±0.248)
		Center 3	0.953	0.781 (±0.759)	0.915	0.681 (±0.728)
		**Mean**	**0.951**	**0.673 (±0.684)**	**0.957**	**0.587 (±0.518)**
	Flare	Center 1	0.986	0.480 (±0.746)	0.999	0.177 (±0.089)
		Center 2	0.993	0.333 (±0.212)	0.997	0.243 (±0.252)
		Center 3	0.998	0.388 (±0.320)	0.996	0.258 (±0.285)
		**Mean**	**0.992**	**0.398 (±0.423)**	**0.997**	**0.239 (±0.236)**
	Roll	Center 1	0.951	0.333 (±0.327)	0.986	0.250 (±0.187)
		Center 2	0.997	0.100 (±0.000)	0.998	0.111 (±0.078)
		Center 3	0.992	0.300 (±0.262)	0.994	0.190 (±0.173)
		**Mean**	**0.980**	**0.208 (±0.204)**	**0.993**	**0.183 (±0.152)**
Right ramus Rotation (degrees)	Auto	Center 1	0.984	1.006* (±0.828)	0.992	0.413 (±0.558)
		Center 2	0.946	0.318* (±0.242)	0.961	0.359 (±0.347)
		Center 3	0.929	0.543 (±0.402)	0.972	0.269 (±0.188)
		**Mean**	**0.953**	**0.538 (±0.524)**	**0.975**	**0.348 (±0.351)**
	Flare	Center 1	0.993	0.560 (±0.464)	0.999	0.263 (±0.176)
		Center 2	0.866	0.886 (±0.795)	0.994	0.185 (±0.188)
		Center 3	0.989	0.450 (±0.372)	0.992	0.330 (±0.203)
		**Mean**	**0.949**	**0.622 (±0.599)**	**0.995**	**0.271 (±0.193)**
	Roll	Center 1	0.999	0.250 (±0.243)	0.998	0.167 (±0.225)
		Center 2	0.935	0.422 (±0.427)	0.994	0.144 (±0.113)
		Center 3	0.981	0.260 (±0.207)	0.996	0.110 (±0.099)
		**Mean**	**0.972**	**0.308 (±0.313)**	**0.996**	**0.142 (±0.135)**

SD = standard deviation, RL = right-left, AP = anteroposterior, SI = superoinferior. Center 1 = Amsterdam UMC, Center 2 = MKA Kennemer&Meer, Center 3 = Radboudumc,

* = statistically significant difference.

The mean inter-observer and intra-observer translational and rotational differences of the maxilla and mandible were all below 0.3 mm and 0.5 degrees. The least observer dependent was the anteroposterior translation of the mandible, for which an inter-observer and intra-observer ICC of 0.996 and 0.999, respectively, were found (Table 4). The differences between the centers were non-significant, except for the inter-observer difference of the yaw of the maxilla (p = 0.047) and the intra-observer difference of the autorotation of the right ramus (p = 0.046).

Table 5 provides the results of the chin analysis. Concerning the translational differences of the chin, the superoinferior direction was slightly more user dependent than the anteroposterior and right-left directions (0.251 mm versus 0.213 mm and 0.223 mm, respectively). The highest difference between users was reported in the pitch with 0.654 degrees inter-observer and 0.604 degrees mean intra-observer difference.

With regard to the left and right ramus, the autorotation of the left ramus and the flare of the right ramus were reported to be most user dependent (with maximal errors of 0.673 degrees and 0.622 degrees). Also, the reported inter- and intra-observer ICCs were all above the 0.94.

## Discussion

The OGA 2.0 presented in the current study is a successor of the OGA presented in an earlier study [11]. Drawbacks of the previous OGA version were the absence of the possibility to assess the postoperative accuracy of the osseous chin, the dependence on a specific virtual planning software and the need for SBM for accurate matching of the rami. In the newly presented tool, the postoperative accuracy of the rami is assessed using VBM instead of SBM. Next to that, the postoperative accuracy of the osseous chin can be assessed and the OGA 2.0 is no longer dependent on any planning software and can be used as a stand-alone program. The OrthoGnathicAnalyser 2.0 is developed to objectively quantify the movements of the individual segments of orthognathic surgery.

### Validation results

The results of this multicenter validation study demonstrated a good reproducibility of the calculated results, with a maximum translational error of 0.26 mm and rotational error of 0.67 degrees, and corresponding high ICCs (>0.92). The current results of the maxilla were comparable to the results described in literature, with an inter-observer and intra-observer ICC of >0.97 and >0.98, for translation and rotation respectively [11, 17]. Stokbro and Thygesen used VBM for measuring the movements of the maxilla and found high ICC values similar to this current study [18]. The translational and rotational results of the mandible showed excellent reproducibility (ICC>0.99 and ICC>0.95 respectively) and were also comparable to previous results [11]. For the different centers, only the inter-observer difference of the yaw of the maxilla (p = 0.047) and the intra-observer difference of the autorotation of the right ramus (p = 0.046) were statistically significant. These differences where however below 0.7 degrees and were therefore considered clinically insignificant. The OGA 2.0 is a robust tool as minimal differences between the centers, and thus different manufacturers of scanners, were reported.

For the matching of the chin, preliminary tests were executed to evaluate which registration technique would perform best. During these tests it was observed that voxel-based matching resulted in less accurate alignment in the sagittal plane due to a deviation in the pitch. It was hypothesized that the result of the voxel-based matching algorithm was affected by the combination of the relatively small volume of the chin and the high-density fixation material. For this reason, it was chosen to implement SBM instead of VBM. This has resulted in a reproducible evaluation of the deviations of the chin segment, with low intra-observer and inter-observer differences (below 0.25 mm or 0.7 degrees). As these results for the analysis of the osseous chin are clinically acceptable it is worth noting that the inter-observer difference for the chin is systematically higher than the maxilla, mandible and rami. Underlying reason for this higher inter-observer difference could be the use of SBM, which required more input of the user.

### Advantages current method

In our previous study [11], the matching of the left and right ramus was performed with SBM to counteract the image artifacts as a result of the sagittal split osteotomy. This technique has resulted in observer differences of more than one degree. Because of the reported difference and the described user dependency in literature [6], the matching technique of the rami was changed to voxel-based matching in OGA 2.0 as there was an updated version of the voxel-based algorithm available. Without correcting the aforementioned image artifacts, the reported maximum error was almost halved to 0.6 degrees. Using VBM instead of SBM is more reproducible which is in line with the findings of Almukhtar et al. [6]. It is also more time-efficient as the input from the user is minimized as the user only selects a ROI instead of manually coloring the surface on which the registration should be performed.

In the previously validated OGA, three landmarks for each jaw segment were required to construct a virtual triangle to allow for the calculation of the clinically relevant translational and rotational movements. Multiple landmark identification has been eliminated by voxel-based registration of the jaw segments. In the new version of OGA, a total of four landmarks needs to be identified instead of the twelve (three for each segment) in the previous OGA version. Identification of only these four landmarks still enables the computation of the required calculations. As a consequence, the workflow becomes more efficient and further eliminates the inaccuracies as a result of multiple landmark identification [19].

The analysis of the chin segment is an important addition in the OGA 2.0. With an easy and reproducible chin segment analysis, studies towards the accuracy or relapse of genioplasty will be more accessible. Furthermore, the added value of using sawing and drilling guides in genioplasty can be objectified.

### Study limitations

The error caused by identification of the landmarks ranged from 0.02 to 2.47 mm [19–21]. Ideally, the manual identification step would be completely eliminated in the software. A promising development is the automatic 3D landmarking using artificial intelligence. Some recent studies have reported errors below 2 mm [22, 23], making automatic 3D landmarking a potential alternative. However, as the landmarks are not used for matching but only function as rotation points, the identification of the landmarks has become of little concern. The high ICCs and low intra- and inter-observer variations support this statement.

The results indicated that the pitch of the chin was still relatively more user dependent than the other variables. It should be explored whether the voxel-based matching method could be adapted to facilitate selection of greyscale values (i.e. selection of the upper threshold) or reorientation of the ROI box to enable exclusion of the high-density fixation material.

For the assessment of the accuracy of the mandible it is important that the postoperative (CB)CT scan was acquired with a correct postoperative occlusion, with relaxed mandibular muscles. For this retrospective study, some scans were acquired in a suboptimal occlusion, which led to an overestimation of the discrepancy in the planned and postoperative outcome. Since the main goal of this study was to validate the novel software, it was chosen not to analyze the surgical outcomes and focus on the validation of the software. For any clinical study, it is imperative to provide proper instruction to the patient before the postoperative scan in order to be able to accurately assess the surgical outcome of the mandible.

## Conclusion

In conclusion, the reported results of this study demonstrated an excellent reproducibility (ICC of >0.92) of the quantification of the skeletal movements between two (CB)CT sets by the OrthoGnathicAnalyser 2.0. By implementing the chin analysis in this software tool, all surgical bony segments can now be objectively evaluated and compared to the preoperative virtual plan. The OrthoGnathicAnalyser 2.0 allows an increased number of evaluations of orthognathic procedures.

## Supporting information

S1 Data (XLSX)

S1 Video (ZIP)
